# Fibromyalgia Concomitant With Rheumatic Diseases in the Eastern Province of Saudi Arabia: A Cross-Sectional Study

**DOI:** 10.7759/cureus.72970

**Published:** 2024-11-04

**Authors:** Khawla K Alghanim, Raghad Alsaidalani, Hoda Al Somali, Hesham Waaer

**Affiliations:** 1 Internal Medicine, King Fahad Military Medical Complex, Dhahran, SAU

**Keywords:** connective tissue diseases, fibromyalgia, osteoarthritis, rheumatic disease, saudi arabia

## Abstract

Objectives: Fibromyalgia (FM) is a complex disorder involving pain dysregulation that can occur independently or alongside other rheumatological illnesses, complicating symptom interpretation. Consequently, this study aimed to evaluate the prevalence of secondary FM in patients with rheumatological diseases and its impact using the Revised Fibromyalgia Impact Questionnaire.

Methods: This cross-sectional descriptive study was conducted at the Rheumatology Outpatient Department of King Fahad Military Medical Complex, Dhahran, from August 2023 to October 2023. Patients with primary rheumatological diseases other than FM were recruited. Secondary FM was diagnosed based on the 2016 revisions of the 2010/2011 FM diagnostic criteria. Data on the demographics of patients were collected through self-reported questionnaires.

Results: Overall, 158 women (76%) and 50 men (24%) with a mean age of 45.7 (12.8) years participated in the study. Systemic lupus erythematosus was the primary diagnosis for most patients. Fisher's exact test (p=0.007) indicated a significant but weak association between osteoarthritis and FM. A family history of comorbidity was significantly associated with an FM diagnosis (p<0.043). Having a major life event, such as the death of a kin, was significantly associated with FM (p<0.032). A personal history of functional disorders was significantly linked to a diagnosis of concomitant FM.

Conclusion: Concomitant FM is frequently undetected in patients with rheumatic diseases. Raising awareness of FM through educational programs may facilitate earlier diagnosis and more effective treatment.

## Introduction

Fibromyalgia (FM) is a complex disorder involving pain dysregulation characterized by chronic fatigue, widespread musculoskeletal pain without evidence of inflammation or structural alterations, hyperalgesia, allodynia, sleep disturbances, and mood disorders [1]. The estimated prevalence of FM is 2-9% worldwide, with a women-to-men ratio of 3:1, and it increases with age [2]. Sex-based differences in the prevalence of FM are attributed to differences in responses to pain, with greater pain sensitivity and higher risk of clinical pain commonly observed among women than among men. Moreover, men are less likely to consult a specialist for chronic pain symptoms, resulting in a lower FM diagnosis rate among men than among women [3].

FM was initially described in the 20th century as inflammation of the fibrous connective tissue of the muscles in certain points of the body named the tender points, which was believed to be the underlying pathophysiology. Repeated attempts to obtain histopathological evidence of inflammation have been unsuccessful [4]. Currently, evidence has shown that dysregulation of the pain processing mechanisms of the central nervous system and peripheral pain generators are responsible for most FM symptoms [4]. Genetics, psychosocial stressors, oxidative stress, and changes in the gut microflora may all have a role in the pathophysiology of FM [5,6].

FM is generally considered a diagnosis of exclusion as no gold standard for its radiological and laboratory diagnosis or otherwise has currently been established. Moreover, the available treatment options are of limited effectiveness. Many different classification criteria for FM have been developed over the years. The first was developed by the American College of Rheumatology (ACR) in 1990 [7-9], which involved widespread pain and tender point examination. However, it was criticized because of the highly examiner-dependent tender point examination and for not including commonly associated symptoms, such as fatigue or sleep disturbance. The subsequent classification criteria developed in 2010/2011 by the ACR disregarded tender point examination and redefined FM as a multi-symptom disorder, including core symptomatology other than musculoskeletal pain, and it was later modified in 2016 to emphasize the generalized nature of the pain and prevent the misclassification of regional pain syndromes. In 2013, ACTION-APS pain taxonomy (AAPT) criteria were developed for FM, which were simplified and modified in 2019 [7-9]. A few studies have compared the 2019 AAPT criteria to the 2016 revised ACR criteria, most of which are in favor of the ACR criteria [10].

FM is a complex, multi-symptom disorder with no biomarkers to assess its activity. Several questionnaires have been developed to determine treatment goals and responses to therapy. For example, the Fibromyalgia Impact Questionnaire (FIQ) is an extensively validated FM-specific tool that captures the overall effect of FM symptomatology, which was developed by Burckhardt et al. in 1991 and revised in 2009 (FIQR) to address certain shortcomings after 18 years of clinical experience with the original version [11,12]. The Fibromyalgia Assessment Status is a valid three-item instrument (pain, fatigue, and sleep disturbances) that performs at least as well as the FIQ in patients with FM and is easier to administer and score. Both questionnaires may be useful for screening patients with FM, with the choice of the most appropriate instrument depending on the setting [13]. FM can occur independently of any other disease or concomitantly with other rheumatological illnesses, making it more difficult to interpret whether symptoms are due to active inflammation or secondary FM, which, in turn, leads to higher reported disease activity and unnecessary escalation of treatment for the primary rheumatological disorder.

This study aimed to evaluate the frequency of secondary FM in patients of rheumatology clinics, assess the impact of demographic factors on the development of concomitant FM, explore the effect of secondary FM on the treatment of primary rheumatological disorders, and assess the impact of FM on patients using the FIQR.

## Materials and methods

This is a cross-sectional descriptive study performed in the Rheumatology Outpatient Department of King Fahad Military Medical Complex (KFMMC), Dhahran, from August 2023 to October 2023. All patients who visited the Rheumatology Outpatient Clinic for a primary rheumatological disease other than FM and fulfilled the inclusion criteria were recruited. The inclusion criteria were as follows: patients aged ≥18 years who agreed to participate in the study in accordance with the Declaration of Helsinki, could read and write Arabic, had physical and cognitive ability to communicate, fulfilled the corresponding criteria for the diagnosis of their primary rheumatological disorder, and had a disease history for >1 year. The exclusion criteria were patients who declined to participate in the study, those with psychiatric or cognitive diseases that impaired the ability to comprehend written or spoken Arabic, and those with comorbid terminal illnesses.

Data on the demographics of patients were collected, including age, sex, marital status, body mass index, education, occupation, comorbid illnesses, family history, medications, smoking, exercise, and major life events.

The 2016 revised ACR criteria for FM were used for FM diagnosis. Patients who fulfilled the criteria were asked to complete the Arabic version of the FIQ (FIQ-A) to assess the impact of FM on patients. The FIQ-A is reliable and valid for assessing the health status and physical function of Arabic-speaking patients with FM [14].

Ethics approval and consent to participate

Patients provided a signed informed consent form after receiving an explanation that their data would be used in this study while maintaining confidentiality. This study was approved by the Armed Forces Hospitals Eastern Province Institutional Review Board (IRB) (approval number: AFHER-IRB-2024-013).

Statistical analysis

All statistical analyses were performed using IBM SPSS Statistics for Windows, Version 21.0 (Released 2012; IBM Corp., Armonk, New York, United States). Categorical variables are presented as frequencies and percentages. Continuous variables were checked for normality of distribution using the Shapiro-Wilk test and are presented as mean (standard deviation) or medians accordingly. Correlations between categorical variables were examined using the chi-squared or Fisher's exact tests as appropriate. Statistical significance was set at a p-value of <0.05.

## Results

Between August 2023 and October 2023, 235 patients were recruited, of which 27 were excluded due to incomplete data. The remaining patients comprised 158 women (76%) and 50 men (24%), with a mean age of 45.7 (12.8). Most patients were non-smokers (185, 88%), married (174, 82%), and from the eastern province of Saudi Arabia (185, 88%). Based on educational level, the patients were grouped into low, including primary school level of education or lower (n=44; 21%); intermediate, including up to high school (n=95; 46%); and high, including undergraduate and postgraduate levels of education (n=69; 33%). The mean disease duration of the patients was 4.9 years (minimum of one year, maximum of 42 years). Most patients (122, 59%) live a sedentary lifestyle, with a reported zero hours of exercise per week. Patients were asked about the presence of any major life events, including but not limited to road traffic accidents, death of kin, physical or emotional abuse, and divorce or separation. Among all patients, 22% reported the presence of major life events, of which the recent death of kin accounted for the majority. The presence of a major life event was significantly associated with FM (p=0.032). Demographic data were further subdivided according to the presence of newly diagnosed concomitant FM and are presented in Table 1.

**Table 1:** Demographic and clinical characteristics of the study participants FM: fibromyalgia; significant p-value <0.05, chi-squared test

Demographic and clinical characteristics of the study participants	
Age years mean (min-max) with FM	48.50 (22-24)
Age years mean (min-max) without FM	45.43 (18-78)
Age total (min-max)	45.7 (12.8)
Sex N (%) with FM female	15 (83%)
Sex N (%) with FM male	3 (17%)
Sex N (%) without FM female	143 (75%)
Sex N (%) without FM male	47 (25%)
Smoking N (%) with FM	1 (6%)
Smoking N (%) without FM	22 (12%)
Originally from the eastern province with FM N (%)	13 (72%)
Originally from the eastern province without FM N (%)	172 (91%)
Married with FM N (%)	16 (89%)
Married without FM N (%)	158 (83%)
Single with FM N (%)	1 (6%)
Single without FM N (%)	15 (8%)
Divorced with FM N (%)	0 (0%)
Divorced without FM N (%)	9 (5%)
Widowed with FM N (%)	1 (6%)
Widowed without FM N (%)	8 (4%)
Disease duration years median (min-max) with FM N (%)	2 (1-15)
Disease duration years median (min-max) without FM N (%)	4 (1-42)
Body mass index mean +/- SD with FM	31.1 (+/- 7)
Body mass index mean +/- SD without FM	29.0 (+/- 7)
Education level low with FM N (%)	7 (39%)
Education level low without FM N (%)	37 (19%)
Education level intermediate with FM N (%)	5 (28%)
Education level intermediate without FM N (%)	90 (47%)
Education level high diagnosed with FM N (%)	6 (33%)
Education level high without FM N (%)	63 (33%)
Exercise per week 0 hours with FM	12 (67%)
Exercise per week 0 hours without FM	110 (57%)
Exercise per week <2 hours with FM	3 (17%)
Exercise per week <2 hours without FM	44 (23%)
Exercise per week 2-4 hours with FM	2 (11%)
Exercise per week 2-4 hours without FM	26 (14%)
Exercise per week >4 hours with FM	1 (6%)
Exercise per week >4 hours without FM	10 (5%)
Presence of major life events with FM	8 (44%)
Presence of major life events without FM	38 (20%)
Recent death of kin among FM	3 (17%), p<0.032
Recent death of kin among without FM	34 (18%)

The primary rheumatological diseases included rheumatoid arthritis (RA) (n=41); spondyloarthropathies (SPA) (n=13); connective tissue disease (n=114); systemic lupus erythematosus (SLE) (n=63); Sjogren's syndrome (n=3); scleroderma (SS) (n=4); other undifferentiated connective tissue diseases (UCTD) (n=44), such as dermatomyositis, polymyositis overlap, and rhesus; vasculitis (n=11); crystal arthropathies (n=11); and osteoarthritis (OA) (n=16). A total of 18 (9%) patients fulfilled the 2016 revised ACR diagnostic criteria for FM, including five patients with OA, two with RA, four with SLE, one with Sjogren, one with SS, one with SPA, and four with UCTD. The results of Fisher's exact test (p=0.007, alpha value <0.05) indicated a significant but weak association between OA and FM (phi coefficient=0.232).

The impact of concomitant FM on patients with primary rheumatological disease was evaluated, and the results showed that FM had a higher impact on patients with SLE as the primary underlying disease in their activities of daily living than on those with other diseases, with a mean FIQR of 65.50 [8] (Table 2). Patients diagnosed with concomitant FM reported a higher frequency of daily use of analgesia (p=0.008, phi coefficient=0.183).

**Table 2:** Prevalence of concomitant fibromyalgia among patients with primary rheumatological disease and the average Fibromyalgia Impact Questionnaire score Fisher's exact test significant p-value <0.05 NS: nonsignificant fibromyalgia; FIQR: Revised Fibromyalgia Impact Questionnaire; RA: rheumatoid arthritis; SPA: spondyloarthropathies; CTD: connective tissue diseases; SLE: systemic lupus erythematosus; OA: osteoarthritis

Rheumatological disease		Fisher's exact P
RA N (%)	41 (19%)	
FM among RA N (%)	2 (4%)	NS
FIQR mean (+/- SD) of patients with RA and FM	54.25 (+/- 6)	
SPA N (%)	13 (6.2%)	
FM among SPA N (%)	1 (8%)	NS
FIQR mean (+/- SD) of patients with SPA and FM	44.67	
SLE N (%)	63 (30%)	
FM among SLE (%)	4 (6%)	NS
FIQR mean (+/- SD) of patients with SLE and FM	60.35 (+/- 14)	
Sjogren N (%)	3 (1%)	
FM among Sjogren N (%)	1 (33%)	NS
FIQR mean (+/- SD) of patients with Sjogren and FM	52.00	
Scleroderma N (%)	4 (2%)	
FM among scleroderma N (%)	1 (25)	NS
FIQR mean (+/- SD) of patients with scleroderma and FM	57.00	
Vasculitis N (%)	11 (5%)	
FM among vasculitis N (%)	nil	NS
FIQR mean (+/- SD) of patients with vasculitis among FM		
Crystal arthropathies N (%)	11 (5%)	
FM among crystal arthropathies N (%)	nil	NS
FIQR mean (+/- SD) of patients with crystal arthropathies among FM		
OA N (%)	16 (8%)	
FM among OA	5 (31%)	P 0.007/phi 0.232
FIQR mean (+/- SD) of patients with OA and FM	51.69 (+/-21)	
Other CTD N (%)	44 (21%)	
FM among other CTD N (%)	4 (9%)	NS
FIQR mean (+/- SD) of patients with other CTD and FM	58.13 (+/-21)	

The relationship between personal and family history of selected comorbidities and FM was assessed. Personal history of depression, anxiety, migraine, irritable bowel syndrome, functional dyspepsia, and obstructive sleep apnea were significantly associated with the diagnosis of concomitant FM. A family history of functional disorders was significantly associated with the diagnosis of FM (p<0.05) with varying degrees of correlation. A family history of comorbidities in general, as well as a family history of diabetes, hypertension, and irritable bowel syndrome, was significantly associated with FM (Table 3).

**Table 3:** Personal and family history of selected comorbidities and fibromyalgia Fisher's exact significant p-value <0.05 NS: nonsignificant; DM: diabetes mellitus; FM: fibromyalgia; HTN: hypertension; IBS: irritable bowel disease; OSA: obstructive sleep apnea

Comorbidity	Personal history	Fisher's exact P/phi coefficient	Family history	Fisher's exact P/phi coefficient
DM N (%)	41 (18%)		83 (44%)	
DM among FM N (%)	2 (5%)	NS	14 (16%)	P 0.006/phi 0.192
HTN N (%)	44 (21%)		70 (34%)	
HTN among FM N (%)	6 (14%)	NS	12 (17%)	P 0.021/phi 0.172
Thyroid disease N (%)	23 (11%)		20 (10%)	
Thyroid disease among FM N (%)	3 (13%)	NS	1 (5%)	NS
Depression N (%)	4 (2%)		8 (4%)	
FM among depression N (%)	2 (50%)	P 0.029/phi 0.151	2 (25%)	NS
Anxiety N (%)	6 (3%)		5 (2%)	
FM among anxiety N (%)	2 (33%)	P 0.029/phi 0.151	0 (%)	NS
Migraine N (%)	14 (7%)		17 (8%)	
FM among migraine N (%)	5 (36%)	P 0.015/phi 0.199	6 (35%)	P 0.001/phi 0.283
IBS N (%)	18 (9%)		27 (13%)	
FM among IBS N (%)	7 (39%)	P 0.002/phi 0.254	7 (26%)	P 0.003/phi 0.237
Functional dyspepsia N (%)	7 (3%)		11 (5%)	
FM among functional dyspepsia N (%)	4 (57%)	P 0.002/phi 0.296	3 (27%)	NS
OSA N (%)	4 (1.9%)		2 (1%)	
FM among OSA N (%)	4 (100%)	P 0.002/phi 0.294	1 (50%)	NS

## Discussion

To our knowledge, data on the prevalence of concomitant FM among patients with rheumatic diseases within Saudi Arabia is scarce. This study evaluated the frequency of secondary FM in patients who visited rheumatology clinics and assessed the impact of demographic factors on the development of concomitant FM.

Overall, 8% of the patients in this study were diagnosed with concurrent FM based on the 2016 revision of the 2010/2011 ACR criteria for FM, which is lower than the prevalence of FM in the general Saudi Arabian population of 13.4% reported by Bawazir [15].

Furthermore, a monocentric study conducted in France assessed the frequency of concurrent FM among patients with rheumatic diseases and reported a prevalence of 8-14% [16]. However, AlOmair et al. [17] reported that 15% of patients with seropositive RA had concomitant FM. This difference can be explained by variances in sample sizes.

Regarding the demographics, most patients with concomitant FM were aged >45 years, similar to the report of AlOmair et al. in which 83% of patients with FM were aged >50 years [17].

Similar to the results of this study, most studies have reported a higher prevalence of primary and secondary FM in women than in men due to differences in their responses to pain, with greater pain sensitivity and higher risk of clinical pain commonly observed among women than among men. Moreover, compared to women, men are less likely to go to a specialist for chronic pain symptoms, resulting in a lower FM diagnosis rate [3].

Most patients (59%) in this study lived a sedentary lifestyle, which was also observed in a recent study, suggesting that patients with FM have higher levels of sedentary lifestyles and worse cardiovascular health outcomes than healthy participants without FM [18].

Experiencing a major life event was significantly associated with FM in our study, with the most common being the death of a kin. This is similar to the results of a study by Kaleycheva et al., in which a significant association was observed between stressor exposure and FM in adults, with the strongest associations observed for physical abuse [19].

Moreover, patients diagnosed with concomitant FM reported a higher frequency of daily use of over-the-counter analgesia despite the lack of strong evidence on the effectiveness of oral non-steroidal anti-inflammatory drugs or opioids in the treatment of FM [20,21]. Therefore, raising awareness toward FM and the use of optimal treatment options to avoid unwanted side effects of analgesia overuse is important.

Personal history of depression, anxiety, migraine, irritable bowel syndrome, functional dyspepsia, and obstructive sleep apnea were significantly associated with the diagnosis of concomitant FM. This is supported by the results of a case-control study conducted by Cole et al., in which the prevalence of FM diagnosed by physical examination was significantly higher among people with (12.9%) than among those without (4.7%) irritable bowel syndrome [22]. Notably, one in five (20%) patients with FM was diagnosed with migraine, and approximately one in 10 patients with chronic migraine was diagnosed with FM (9.9%) [23].

Several studies have addressed the frequency of FM in patients with a family history of FM. In one study, among the 58 offspring of 20 complete nuclear families with mothers diagnosed with FM, 16 (28%) were found to have FM. Since exposure to environmental factors, as well as psychological and familial factors, did not differ between offspring with and without FM, the higher familial occurrence of the condition might be attributable to genetic factors [24]. Another study showed that patients with FM exhibited higher rates of familial major mood disorders, as well as higher lifetime rates of migraine, irritable bowel syndrome, chronic fatigue syndrome, major depression, and panic disorder [25], as mentioned in our study.

This study has some limitations. Since the questionnaire depends on the recall ability of patients, recall bias could exist. The limited sample size and cross-sectional design of the study make it difficult to estimate associations precisely, hence highlighting the need for larger studies targeting the frequency of concurrent FM in patients with rheumatic disease.

## Conclusions

The findings of our study suggest that FM is frequently overlooked in patients with rheumatic diseases, and thus, the reported prevalence is lower than that in the general population. Additionally, we observed a higher likelihood of FM in women and older individuals, as well as patients with sedentary lifestyles and a history of other illnesses or family members with FM. Implementing educational programs to raise awareness on FM can help improve its diagnosis and treatment.
